# Antithrombin use and mortality in patients with stage IV solid tumor-associated disseminated intravascular coagulation: a nationwide observational study in Japan

**DOI:** 10.1186/s12885-020-07375-2

**Published:** 2020-09-09

**Authors:** Kohei Taniguchi, Hiroyuki Ohbe, Kazuma Yamakawa, Hiroki Matsui, Kiyohide Fushimi, Hideo Yasunaga

**Affiliations:** 1 Translational Research Program, Osaka Medical College, 2-7 Daigaku-machi, Takatsuki, Osaka, 569-8686 Japan; 2 Department of Clinical Epidemiology and Health Economics, School of Public Health, The University of Tokyo, 7-3-1 Hongo, Bunkyo-ku, Tokyo, 113-0033 Japan; 3 Department of Emergency Medicine, Osaka Medical College, 2-7 Daigaku-machi, Takatsuki, Osaka, 569-8686 Japan; 4 Department of Health Policy and Informatics, Tokyo Medical and Dental University Graduate School of Medicine, 1-5-45 Yushima, Bunkyo-ku, Tokyo, 113-8510 Japan

**Keywords:** Anticoagulant, Antithrombin, Disseminated intravascular coagulation, Mortality, Solid tumor

## Abstract

**Background:**

Terminal-stage solid tumors are one of the main causes of disseminated intravascular coagulation (DIC); effective therapeutic strategies are therefore warranted. This study aimed to investigate the association between mortality and antithrombin therapy in patients with stage IV solid tumor-associated DIC using a large nationwide inpatient database.

**Methods:**

From July 2010 to March 2018, patients with stage IV solid tumor-associated DIC in the general wards, intensive care unit, or high care unit were identified using the Japanese Diagnosis Procedure Combination Inpatient Database. Patients who received antithrombin within 3 days of admission were allocated to the antithrombin group, while the remaining patients were allocated to the control group. One-to-four propensity score matching analyses were applied to compare outcomes. The primary outcome was the 28-day in-hospital mortality.

**Results:**

Of the 25,299 eligible patients, 919 patients had received antithrombin within 3 days of admission and were matched with 3676 patients in the control group. There were no significant differences in the 28-day mortality between the two groups (control vs. antithrombin: 28.9% vs. 30.3%; hazard ratio, 1.08; 95% confidence interval, 0.95–1.23). There were no significant differences in the organ failure score and the proportion of critical bleeding between the two groups. Subgroup analyses showed that the effects of antithrombin were not significantly different among different tumor types.

**Conclusion:**

Using a nationwide Japanese inpatient database, this study showed that there is no association between antithrombin administration and 28-day mortality in patients with stage IV solid tumor-associated DIC. Therefore, establishing other therapeutic strategies for solid tumor-associated DIC is required.

## Background

Chronic hypercoagulable states are present in patients with cancer, especially those with terminal (stage IV) cancers [1]. Besides venous thromboembolism, cancers cause disseminated intravascular coagulation (DIC), an extreme hypercoagulable state [2]. The hallmark of DIC is the activation of systemic intravascular coagulation and subsequent consumption of coagulation-related proteins and thrombocytes, resulting in vascular thrombotic occlusion and hemorrhagic complications [3]. There are multiple underlying causes of DIC; among them, solid tumor-associated DIC accounts for a quarter of all cases [4, 5]. Among patients with solid tumors, various comorbid factors, such as infection or chemotherapy, could possibly induce DIC [6]. It has been indicated that the survival was lower in patients with solid tumors who developed DIC than in those who did not [7]. The cornerstone of DIC management is treatment of the underlying disorder through surgery or through chemotherapy in patients with cancer [8]. However, in the terminal stages of solid tumors, surgical resection is not always possible, and the treating physician may be a reluctant to initiate chemotherapy due to its side effects, such as bone marrow suppression. Therefore, it is often difficult to initiate or continue multimodal cancer treatments [1, 6]. Hence, other supportive therapies are desired in the management of solid tumor-associated DIC.

The essence of DIC is the systemic activation of coagulation. Besides the underlying disease treatment, anticoagulant drugs and/or supplemental coagulation suppressors may be a potent adjuvant therapy. One of the features of DIC is a reduced level of endogenous coagulation suppressors, such as antithrombin (AT), due to the consumption coagulopathy [9]. Reduced levels of AT due to DIC associated consumption coagulopathy determine a hypercoagulable state. Thus, the use of AT concentrate to increase the AT plasma levels may reduce this prothrombotic state. Additionally, AT supplemental therapy may reduce the risk of hemorrhagic complications induced by other anticoagulants, such as heparins [10]. Supplementation of AT is administered due to its anticoagulation and anti-inflammatory effects [8]. However, the effects of AT therapy on DIC are controversial. Previously, some randomized controlled trials and meta-analyses have indicated no beneficial effects of AT therapy in patients with sepsis [11, 12]. There are, however, several reports indicating the positive effects of AT therapy in patients with sepsis-associated DIC [13–15].

Until now, the effects of AT therapy on solid tumor-associated DIC have not been investigated thoroughly. Therefore, this study aimed to evaluate the association between AT therapy and DIC caused by stage IV solid tumors, using a nationwide inpatient database in Japan.

## Methods

### Ethical statement

The protocol of this study was approved by the Institutional Review Board of The University of Tokyo (approval number: 3501–3; December 25, 2017). This study was conducted using routinely collected data. Informed consent was not required because of the anonymous nature of the retrospective data.

### Data source

Data were collected from the Japanese Diagnosis Procedure Combination Inpatient Database. This database contains discharge summaries and administrative claims from more than 1200 acute care hospitals, which accounts for approximately half of all acute admissions in Japan. The database includes data on age, sex, body weight, body height, level of consciousness at admission, diagnoses (main diagnosis, comorbidities present at admission, and complications arising after admission) recorded according to the International Classification of Diseases Tenth Revision (ICD-10) codes, procedures, prescriptions, drug administration, and discharge status. Attending physicians are required to report objective evidence for their diagnoses for the purpose of treatment cost reimbursement, since the payment system and these diagnostic records are linked [16]. A previous validation study of this database has indicated that the specificity of diagnosis for DIC was 98.2% [17].

### Patient selection

All patients diagnosed with DIC (ICD-10 code: D65) from July 1, 2010, to March 31, 2018, in the general wards, intensive care unit, or high care unit were identified. Of these, patients who were admitted with the following stage IV solid tumors were included: esophagus (ICD-10 code: C15), stomach (C16), colon (C18–C20), liver (C22), bile duct/gallbladder (C23, C24), pancreas (C25), lung (C33, C34, C37–C39), breast (C50), gynecological (C53, C54, C56), and urological (C61, C64–C67). Stage IV was defined according to the TNM staging system for each solid tumor or recurrence. We excluded patients (i) younger than 18 years, (ii) admitted with two or more solid tumors, (iii) who were pregnant, (iv) who were admitted for the second or subsequent time with a diagnosis of DIC during the study period, and (v) who were discharged or died within 3 days of admission. Patients who received AT within 3 days of admission were defined as the AT group, while the remaining patients were defined as the control group.

### Covariates and outcomes

The following characteristics were used as covariates: age, sex, body mass index at admission, Japan Coma Scale at admission [18], Charlson Comorbidity Index [19], presence of sepsis at admission, year of admission, teaching hospital, ambulance use, emergency admission, surgery within 3 days of admission, recurrence, type of solid tumor, metastatic condition, examinations within 3 days of admission, and treatments within 3 days of admission. Body mass index was categorized as < 18.5, 18.6–24.9, 25.0–29.9, ≥30.0 kg/m^2^, or missing data. Japan Coma Scale status, which is highly correlated with the Glasgow Coma Scale score, was categorized into alert consciousness, confusion, somnolence, and coma [18]. The Charlson Comorbidity Index, which is scored based on diagnoses for individual patients, was categorized as 0, 1, 2–4, 5–7, or ≥ 8 [19]. We included the following metastatic conditions according to the ICD-10 codes: lung metastasis (ICD-10 code: C780), peritoneal metastasis (C786), liver metastasis (C787), brain metastasis (C793), bone metastasis (C795), and other metastases (C77, C781–C785, C788, C790–C792, C794, and C796–C799).

The 28-day mortality was set as the primary outcome. Organ failure scores and the proportion of critical bleeding were set as secondary outcomes. Organ failure scores (cardiovascular, respiratory, neurologic, hematologic, hepatic, and renal systems) were calculated based on ICD-10 codes or procedure codes within 28 days of admission [20] (listings of the codes are available in Table S1). The criteria for critical bleeding included those who underwent endoscopic hemostasis within 28 days of admission, were diagnosed with respiratory tract bleeding as a complication (ICD-10 code: R042, R048, or R049), were diagnosed with intracranial hemorrhage as a complication (I60, I61, I621, or I629), or received ≥720 ml/day of red blood cells within 28 days of admission.

### Propensity score matching

A propensity score matching method was used to compare outcomes between the two groups [21, 22]. Propensity scores of patients receiving AT within 3 days of admission were predicted by a multivariable logistic regression model with all the covariates in Table 1 as predictive variables. One-to-four nearest-neighbor matching with replacement was conducted for the estimated propensity scores of the patients using a caliper width set at 20% of the standard deviation for the propensity scores [21, 22]. Distribution of propensity scores before and after matching is shown in Figures S1A and B. Each covariate was compared before and after propensity score matching by using absolute standardized differences. Less than 10% of the absolute standardized differences were regarded as denoting negligible imbalances between the two groups [23]. Propensity score matching was conducted using the PSMATCH2 module of the STATA software (Stata Corp., College Station, TX).

**Table 1:** Baseline characteristics before and after propensity score matching

Covariates	Overall cohort			Matched cohort		
	Control (***n*** = 24,377)	AT (***n*** = 922)	ASD	Control (***n*** = 3676)	AT (***n*** = 919)	ASD
**Age, mean (SD)**	69.4 (11.5)	69.3 (11.2)	0.6	69.3 (11.8)	69.3 (11.3)	0.0
**Male, n (%)**	15,271 (62.6%)	498 (54.0%)	17.6	1993 (54.2%)	498 (54.2%)	0.1
**Body mass index, kg/m**^**2**^**, n (%)**						
< 18.5	5459 (22.4%)	194 (21.0%)	3.3	754 (20.5%)	194 (21.1%)	1.5
18.5–24.9	14,135 (58.0%)	541 (58.7%)	1.4	2167 (58.9%)	538 (58.5%)	0.8
25.0–29.9	2888 (11.8%)	99 (10.7%)	3.5	407 (11.1%)	99 (10.8%)	1.0
≥ 30.0	440 (1.8%)	22 (2.4%)	4.1	78 (2.1%)	22 (2.4%)	1.8
Missing	1455 (6.0%)	66 (7.2%)	4.8	270 (7.3%)	66 (7.2%)	0.6
**Japan Coma Scale at admission, n (%)**						
Alert	22,030 (90.4%)	743 (80.6%)	28.0	3006 (81.8%)	741 (80.6%)	2.9
Confusion	1742 (7.1%)	120 (13.0%)	19.6	473 (12.9%)	119 (12.9%)	0.2
Somnolence	442 (1.8%)	35 (3.8%)	12.0	131 (3.6%)	35 (3.8%)	1.3
Coma	163 (0.7%)	24 (2.6%)	15.3	66 (1.8%)	24 (2.6%)	5.6
**Charlson Comorbidity Index, n (%)**						
0	5168 (21.2%)	239 (25.9%)	11.1	975 (26.5%)	239 (26.0%)	1.2
1	3409 (14.0%)	101 (11.0%)	9.2	415 (11.3%)	101 (11.0%)	1.0
2–4	4979 (20.4%)	256 (27.8%)	17.2	1032 (28.1%)	255 (27.7%)	0.7
5–7	8455 (34.7%)	234 (25.4%)	20.4	916 (24.9%)	233 (25.4%)	1.0
≥ 8	2366 (9.7%)	92 (10.0%)	0.9	338 (9.2%)	91 (9.9%)	2.4
**Presence of sepsis at admission, n (%)**	8042 (33.0%)	533 (57.8%)	51.5	2087 (56.8%)	530 (57.7%)	1.8
**Year at admission, year, n (%)**						
2010–2011	6094 (25.0%)	217 (23.5%)	3.4	864 (23.5%)	215 (23.4%)	0.3
2012–2013	6955 (28.5%)	247 (26.8%)	3.9	1005 (27.3%)	246 (26.8%)	1.3
2014–2015	5859 (24.0%)	247 (26.8%)	6.3	976 (26.6%)	247 (26.9%)	0.7
2016–2017	5469 (22.4%)	211 (22.9%)	1.1	831 (22.6%)	211 (23.0%)	0.8
**Teaching hospital, n (%)**	16,569 (68.0%)	670 (72.7%)	10.3	2681 (72.9%)	668 (72.7%)	0.6
**Ambulance use, n (%)**	3612 (14.8%)	278 (30.2%)	37.4	1080 (29.4%)	275 (29.9%)	1.2
**Emergency admission, n (%)**	12,904 (52.9%)	653 (70.8%)	37.5	2613 (71.1%)	650 (70.7%)	0.8
**Any operation within 3 days of admission, n (%)**	1640 (6.7%)	242 (26.2%)	54.5	989 (26.9%)	239 (26.0%)	2.0
**Recurrence**	11,637 (47.7%)	458 (49.7%)	3.9	1866 (50.8%)	457 (49.7%)	2.1
**Type of solid tumor, n (%)**						
Esophagus	721 (3.0%)	16 (1.7%)	8.1	59 (1.6%)	16 (1.7%)	1.1
Stomach	3754 (15.4%)	93 (10.1%)	16.0	370 (10.1%)	92 (10.0%)	0.2
Colorectal	3707 (15.2%)	203 (22.0%)	17.6	742 (20.2%)	202 (22.0%)	4.4
Liver	2751 (11.3%)	127 (13.8%)	7.5	535 (14.6%)	127 (13.8%)	2.1
Bile duct/gallbladder	2344 (9.6%)	107 (11.6%)	6.5	420 (11.4%)	107 (11.6%)	0.7
Pancreas	3627 (14.9%)	163 (17.7%)	7.6	692 (18.8%)	163 (17.7%)	2.8
Lung, trachea, and mediastinum	3182 (13.1%)	198 (10.0%)	33.3	133 (10.8%)	36 (10.0%)	1.6
Breast	799 (3.3%)	28 (3.0%)	1.4	118 (3.2%)	28 (3.0%)	0.9
Gynecological	1107 (4.5%)	95 (10.3%)	22.1	390 (10.6%)	94 (10.2%)	1.2
Urological	2385 (9.8%)	54 (5.9%)	14.7	217 (5.9%)	54 (5.9%)	0.1
**Metastatic condition**						
Lung metastasis	1595 (6.5%)	56 (6.1%)	1.9	207 (5.6%)	56 (6.1%)	2.0
Peritoneum metastasis	3009 (12.3%)	99 (10.7%)	5.0	376 (10.2%)	98 (10.7%)	1.4
Liver metastasis	5410 (22.2%)	160 (17.4%)	12.2	642 (17.5%)	159 (17.3%)	0.4
Brain metastasis	1212 (5.0%)	14 (1.5%)	19.6	49 (1.3%)	14 (1.5%)	1.6
Bone metastasis	4293 (17.6%)	92 (10.0%)	22.3	332 (9.0%)	92 (10.0%)	3.3
Other metastasis	3538 (14.5%)	101 (11.0%)	10.7	390 (10.6%)	101 (11.0%)	1.2
**Examinations or treatments within 3 days of admission, n (%)**						
Intensive or high care unit admission	4212 (17.3%)	285 (30.9%)	32.3	1042 (28.3%)	283 (30.8%)	5.4
Bacterial culture test	6334 (26.0%)	576 (62.5%)	79.0	2306 (62.7%)	574 (62.5%)	0.6
Endoscopy	2160 (8.9%)	48 (5.2%)	14.3	166 (4.5%)	48 (5.2%)	3.3
Computed tomography	11,546 (47.4%)	631 (68.4%)	43.7	2564 (69.7%)	629 (68.4%)	2.8
Oxygen supplementation	5871 (23.3%)	436 (47.3%)	49.9	1724 (44.6%)	435 (44.3%)	0.9
Mechanical ventilation	411 (1.7%)	146 (15.8%)	51.7	557 (15.2%)	144 (15.7%)	1.4
Renal replacement therapy	277 (1.1%)	60 (6.5%)	28.3	179 (4.9%)	59 (6.4%)	6.7
Central venous catheter insertion	2518 (10.3%)	350 (38.0%)	68.2	1353 (36.8%)	347 (37.8%)	2.0
Endoscopic hemostasis	171 (0.7%)	8 (0.9%)	1.9	24 (0.7%)	8 (0.9%)	2.5
Dopamine	1364 (5.6%)	222 (24.1%)	53.8	911 (24.8%)	220 (23.9%)	2.0
Dobutamine	82 (0.3%)	42 (4.6%)	27.6	114 (3.1%)	39 (4.2%)	6.1
Noradrenaline	625 (2.6%)	223 (24.2%)	67.0	820 (22.3%)	220 (23.9%)	3.9
Adrenaline	340 (1.4%)	30 (3.3%)	12.4	137 (3.7%)	29 (3.2%)	3.1
Vasopressin	36 (0.1%)	26 (2.8%)	22.2	90 (2.4%)	25 (2.7%)	1.7
Thrombomodulin	1609 (6.6%)	370 (40.1%)	86.3	1414 (38.5%)	367 (39.9%)	3.0
Tranexamic acid	1050 (4.3%)	52 (5.6%)	6.1	232 (6.3%)	51 (5.5%)	3.2
Serine protease inhibitors	2760 (11.3%)	305 (33.1%)	54.2	1170 (31.8%)	302 (32.9%)	2.2
Heparin	1482 (6.1%)	148 (16.1%)	32.2	605 (16.5%)	146 (15.9%)	1.6
Antiplatelet	521 (2.1%)	18 (2.0%)	1.3	81 (2.2%)	18 (2.0%)	1.7
Anticoagulant	415 (1.7%)	10 (1.1%)	5.3	43 (1.2%)	10 (1.1%)	0.8
Antibiotics	11,125 (45.6%)	807 (87.5%)	99.1	3338 (90.8%)	804 (87.5%)	10.7
Chemotherapy	2343 (9.6%)	45 (4.9%)	18.3	197 (5.4%)	45 (4.9%)	2.1
Molecular targeted therapy	463 (1.9%)	7 (0.8%)	10.0	22 (0.6%)	7 (0.8%)	2
Steroids	5181 (21.3%)	211 (22.9%)	3.9	817 (22.2%)	209 (22.7%)	1.2
Diuretics	3703 (15.2%)	289 (31.3%)	38.9	1124 (30.6%)	287 (31.2%)	1.4
Antiemetic	4475 (18.4%)	134 (14.5%)	10.3	515 (14.0%)	134 (14.6%)	1.6
Non-narcotic analgesics	11,115 (45.6%)	544 (59.0%)	27.1	2221 (60.4%)	543 (59.1%)	2.7
Narcotic	6967 (28.6%)	428 (46.4%)	37.5	1721 (46.8%)	425 (46.2%)	1.1
Parenteral nutrition	1169 (4.8%)	89 (9.7%)	18.8	317 (8.6%)	87 (9.5%)	2.9
Insulin	2694 (11.1%)	217 (23.5%)	33.5	861 (23.4%)	214 (23.3%)	0.3
Red blood cell	3660 (15.0%)	352 (38.2%)	54.3	1426 (38.8%)	350 (38.1%)	1.5
Fresh frozen plasma	1238 (5.1%)	290 (31.5%)	72.6	1162 (31.6%)	287 (31.2%)	0.8
Platelets	1459 (6.0%)	228 (24.7%)	53.8	892 (24.3%)	226 (24.6%)	0.8
Red blood cell ≥720 ml/day	595 (2.4%)	106 (11.5%)	36.1	410 (11.2%)	105 (11.4%)	0.9

*AT* Antithrombin, *SD* Standard deviation, *ASD* Absolute standardized differences

### Statistical analysis

To compare the 28-day mortality between the two groups, a Kaplan–Meier analysis and a Cox proportional hazards regression analysis were conducted after propensity score matching. Patients were excluded based on survival at 28 days after admission. We used the Cox proportional hazards survival methods accompanied by cluster-robust standard errors, with hospitals used as the cluster variable.

Secondary outcomes were assessed through a generalized estimating equation approach accompanied by cluster-robust standard errors, using hospitals as the cluster variable [24]. Odds ratios and their 95% confidence intervals (CIs) were calculated for binary outcomes. Similarly, differences and their 95% CIs were calculated for continuous outcomes. The logit link function was used for odds ratios, and the identity link function was used for differences in the generalized estimating equation approach. As a subgroup analysis, the heterogeneity of the treatment effects on the 28-day mortality for the presence of sepsis at admission and for each type of solid tumor were investigated in the propensity score-matched cohort.

Categorical variables are shown as numbers and percentages, and continuous variables are shown as means and standard deviations (SD). All reported *p*-values were two-sided, and values < 0.05 were considered significant. All analyses were conducted using STATA/MP 16.0 (Stata Corp., College Station, TX, USA).

## Results

A total of 389,658 patients were diagnosed with DIC during the 93-month study period. Of these, 29,453 patients with stage IV solid tumors were included. Finally, 25,299 patients were eligible based on our inclusion criteria. A total of 24,377 patients were categorized into the control group and 922 patients were categorized into the AT group. The mean amount of antithrombin administered in the AT group was 1621 (SD 426) IU daily for 5.2 (SD 9.9) days.

**Fig. 1:**
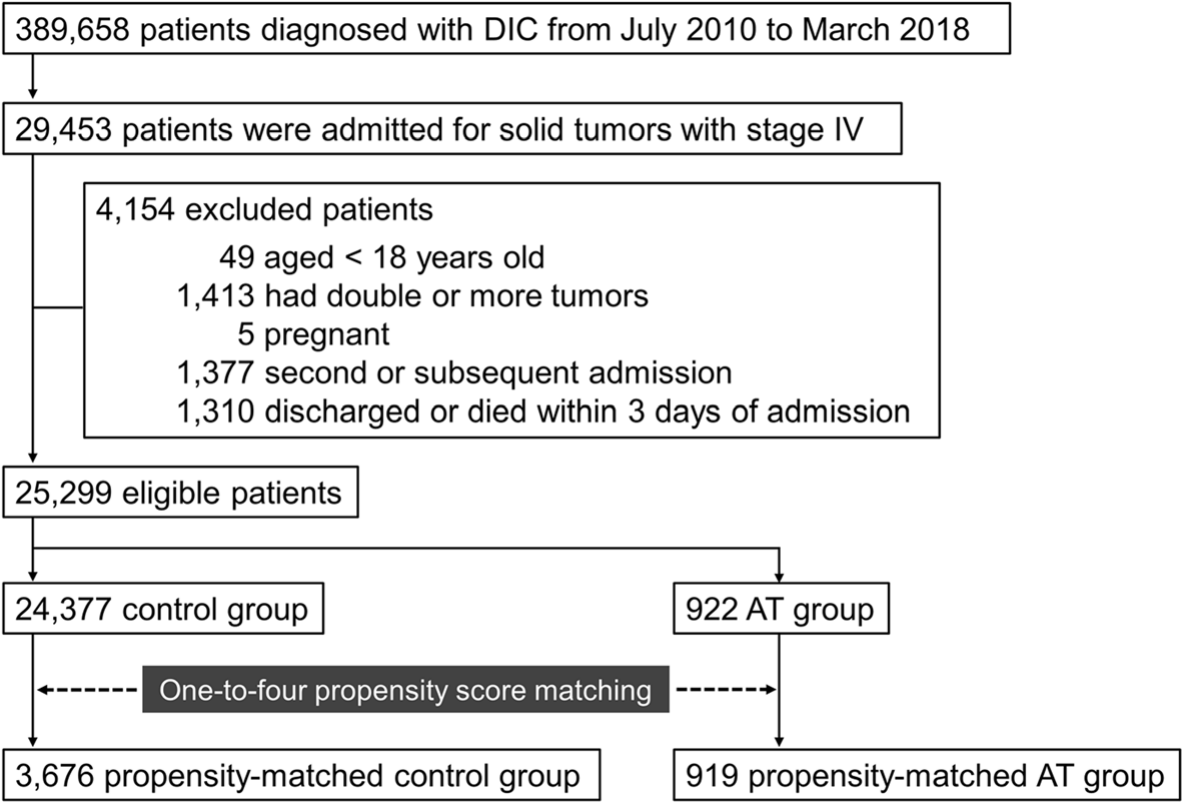
Flowchart of patient selection. DIC, disseminated intravascular coagulation; AT, antithrombin

Table 1 shows the baseline characteristics of the patients before and after propensity score matching. One-to-four propensity score matching created a cohort with a total of 4595 patients, including 3676 patients in the control group and 919 patients in the AT group (Fig. 1). After propensity score matching, the covariates were well balanced between the two groups (Table 1).

**Fig. 2:**
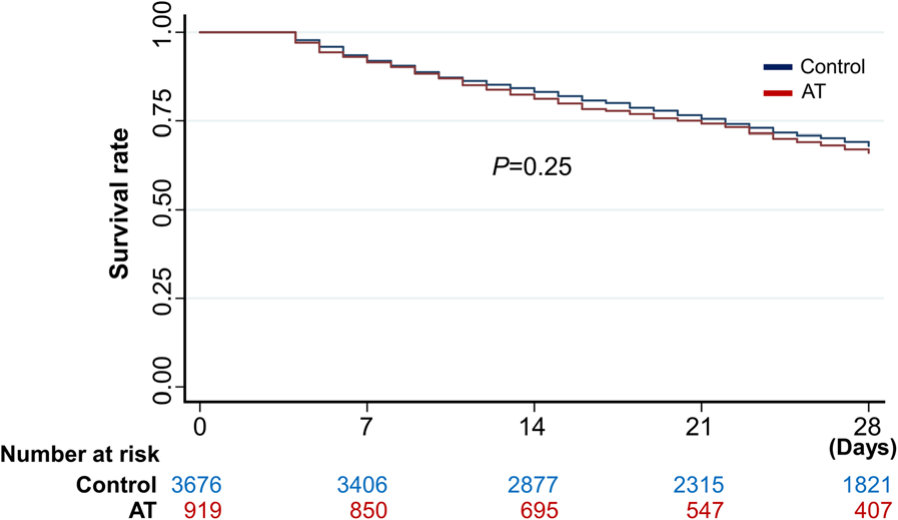
Kaplan–Meier survival plots for stage IV solid tumors associated with disseminated intravascular coagulation, and solid tumors treated with or without antithrombin in propensity-matched groups. There was no significant difference in survival rate between the two groups (*P* = 0.25). AT, antithrombin

The overall 28-day mortality was 30.9% (7823/25,299). Kaplan–Meier analysis and Cox proportional hazards regression analysis showed no significant difference in the 28-day mortality between the two groups in the matched cohort (control vs. AT: 28.9% vs. 30.3%; hazard ratio [HR], 1.08; 95% CI, 0.95–1.23) (Fig. 2 and Table 2). There was no significant difference between the two groups in the organ failure scores (control vs. AT: 1.80 vs. 1.78; difference 0.04; 95% CI, − 0.05–0.12) and in the prevalence of critical bleeding (control vs. AT: 6.9% vs. 6.1%; odds ratio, 0.86; 95% CI, 0.60–1.24).

**Table 2:** Outcomes in the overall and matched cohorts and results of propensity score matching analysis

Outcomes	Unmatched cohort		Matched cohort		Hazard ratios, odds ratios or differences (95% CI)	***P***-value
	Control (***n*** = 24,377)	AT (***n*** = 922)	Control (***n*** = 3676)	AT (***n*** = 919)		
**28-day mortality, n (%)**	7545 (31.0%)	278 (30.2%)	1061 (28.9%)	278 (30.3%)	1.08 (0.95 to 1.23)	0.37
**Organ failure score, mean (SD)**	1.46 (0.71)	1.78 (0.93)	1.80 (0.90)	1.78 (0.93)	0.04 (−0.05 to 0.12)	0.40
**Critical bleeding, n (%)**	1338 (5.5%)	56 (6.1%)	254 (6.9%)	56 (6.1%)	0.86 (0.60 to 1.24)	0.42

*AT* Antithrombin, *CI* Confidence intervals, *SD* Standard deviation

Subgroup analyses showed no significant interactions in the 28-day mortality between the treatment group and the types of solid tumors (Table 3). A significant interaction between AT use and the presence of sepsis at admission on 28-day mortality was observed (*P*-value for interaction = 0.028).

**Table 3:** Subgroup analyses of 28-day mortality

Subgroup	Number of patients	Control	AT	Hazard ratios (95% CI)	***P***-value for interaction
**Esophagus**					
Yes	75	15/59 (25.0%)	6/16 (38.0%)	1.41 (0.55 to 3.64)	0.52
No	4520	1046/3617 (28.9%)	272/903 (30.1%)	1.07 (0.91 to 1.26)	
**Stomach**					
Yes	462	147/370 (39.7%)	35/92 (38.0%)	0.89 (0.60 to 1.33)	0.31
No	4133	914/3306 (27.6%)	243/827 (29.4%)	1.11 (0.93 to 1.32)	
**Colorectal**					
Yes	944	140/742 (18.9%)	40/202 (19.8%)	1.04 (0.70 to 1.55)	0.80
No	3651	921/2934 (31.4%)	238/717 (33.2%)	1.11 (0.93 to 1.31)	
**Liver**					
Yes	662	152/535 (28.4%)	40/127 (31.5%)	1.22 (0.82 to 1.81)	0.52
No	3933	909/3141 (28.9%)	238/792 (30.1%)	1.06 (0.89 to 1.26)	
**Bile duct / gallbladder**					
Yes	527	131/420 (31.2%)	29/107 (27.1%)	0.85 (0.56 to 1.30)	0.19
No	4068	930/3256 (28.6%)	249/812 (30.7%)	1.11 (0.94 to 1.32)	
**Pancreas**					
Yes	855	207/692 (29.9%)	56/163 (34.4%)	1.22 (0.90 to 1.67)	0.39
No	3740	854/2984 (28.6%)	222/756 (29.4%)	1.05 (0.88 to 1.25)	
**Lung, trachea, and mediastinum**					
Yes	169	67/133 (50.4%)	21/36 (58.3%)	1.25 (0.78 to 2.00)	0.46
No	4426	994/3543 (28.1%)	257/883 (29.1%)	1.07 (0.90 to 1.26)	
**Breast**					
Yes	146	50/118 (42.4%)	10/28 (35.7%)	0.82 (0.43 to 1.57)	0.40
No	4449	1011/3558 (28.4%)	268/891 (30.1%)	1.09 (0.93 to 1.29)	
**Gynecological**					
Yes	484	94/390 (24.1%)	21/94 (22.3%)	0.99 (0.52 to 1.89)	0.89
No	4111	967/3286 (29.4%)	257/825 (31.2%)	1.08 (0.92 to 1.27)	
**Urological**					
Yes	271	58/217 (26.7%)	20/54 (37.0%)	1.57 (0.90 to 2.75)	0.16
No	4324	1003/3459 (29.0%)	258/865 (29.8%)	1.05 (0.89 to 1.24)	
**Sepsis at admission**					
Yes	2617	571/2087 (27.4%)	130/530 (24.5%)	0.90 (0.72 to 1.12)	0.028
No	1978	490/1589 (30.8%)	148/389 (38.0%)	1.36 (1.09 to 1.63)	

*AT* Antithrombin, *CI* Confidence intervals

## Discussion

This study examined the association between AT treatment and stage IV solid tumor-associated DIC for the first time by using a large Japanese inpatient database. Our results showed that AT treatment did not improve the 28-day mortality in patients with stage IV solid tumor-associated DIC.

AT inhibits coagulation through factors IIa (thrombin) and Xa [10]. AT also neutralizes other coagulation enzymes such as plasmin, factors IXa, XIa, and XIIa [10, 25]. These effects suggest that AT is an essential regulator in the coagulation cascade [26]. In addition, AT has anti-inflammatory effects through the inhibition of both coagulation-dependent and -independent mechanisms [10, 25]. Furthermore, AT may exert antitumor activity through the suppression of angiogenesis [27]. Based on these pathophysiological mechanisms, we hypothesized that AT may be beneficial for patients with stage IV solid tumor-associated DIC. However, this study did not show improved outcomes in the AT group. Our results may imply that the condition of stage IV solid tumor itself has a stronger effect on mortality than the effects of AT treatment. Another possibility is that only one AT supportive therapy was not enough to show improved outcomes for stage IV solid tumor-associated DIC.

The type of cancer may be an important factor in considering the treatment for cancer-associated DIC. The symptoms of DIC vary depending on the type of cancer. DIC associated with hematological malignancies is categorized as an enhanced-fibrinolytic type and presents mainly with bleeding symptoms, while DIC associated with solid tumors is categorized as a balanced-fibrinolytic type [28]. Among solid tumors, hepatocellular carcinoma, lung cancer, and gastric cancer are more prone to causing DIC [6]. Each type of solid tumor has a different biological mechanism for recurrence and metastasis heterogeneously. Therefore, we assumed that the reaction of each type of solid tumor to AT therapy might be different. However, the results of subgroup analyses in this study showed no heterogeneous effects of AT among different types of solid tumors. These results also suggest that the influence of stage IV tumors alone was extremely significant as compared to the effects of AT treatment.

Other than advanced malignant diseases, sepsis is one of the central underlying causes of DIC occurrence. In the present study, approximately half of the patients had sepsis at admission. The sepsis-induced DIC was classified with organ failure type (hypercoagulation predominance type) [8]; however, the validity of AT therapy has been controversial even in sepsis-associated DIC [29]. However, solid tumor-associated DIC is difficult to classify into any specific DIC type (i.e., bleeding type, organ failure type, and the massive bleeding or consumptive type) [8]. Recently, solid tumor-associated DIC led to an unfavorable outcome through bleeding complications in cancer patients with venous thromboembolism [30], which was consistent with our negative findings.

This study has several limitations. This was a retrospective observational study, and some bias due to unmeasured confounders may still be present. For example, the results of blood tests such as serum AT levels, platelet count, and D-dimer were not available in the current database, and therefore, we could not examine a DIC score and resolution rate of DIC [31]. Further, the dose of AT in this study might not have been enough to show an improved outcome. The Japanese Ministry of Health, Labour and Welfare has approved a supplementary AT dose (1500–3000 IU/day) for patients with DIC based on a previous randomized trial [14]; however, this dosage is markedly lower than that reported in the Kyber-Sept trial (30,000 IU/4 days) [11]. In the present study, this low AT usage trend was maintained (mean; 1621 IU/day), and this dosage may be insufficient for the improvement of stage IV solid tumor-associated DIC. The exact time of onset of DIC was unclear, so some patients in the control group may have developed DIC induced by chemotherapy or infection after admission.

## Conclusions

This large nationwide observational study did not indicate the benefits of AT treatment for stage IV solid tumor-associated DIC. Therefore, establishing other therapeutic strategies for solid tumor-associated DIC is required.

## Supplementary information

**Additional file 1: Figure S1.** Distribution of propensity score (A) Before matching analysis (B) After matching analysis. AT, antithrombin.

**Additional file 2: Table S1.** ICD-10 codes and Japanese procedure codes for organ failure scores.

## Data Availability

The datasets used and/or analyzed during the current study are available from the corresponding author on reasonable request.

## Abbreviations

DIC: Disseminated intravascular coagulation
AT: Antithrombin
ICD-10: International Classification of Diseases Tenth Revision
CIs: Confidence intervals
