# Supplementary figures and images for: Antithrombin use and mortality in patients with stage IV solid tumor-associated disseminated intravascular coagulation: a nationwide observational study in Japan
Source: BMC Cancer. 2020 Sep 9;20:867. doi: 10.1186/s12885-020-07375-2 (PMC7488043; doi:10.1186/s12885-020-07375-2)

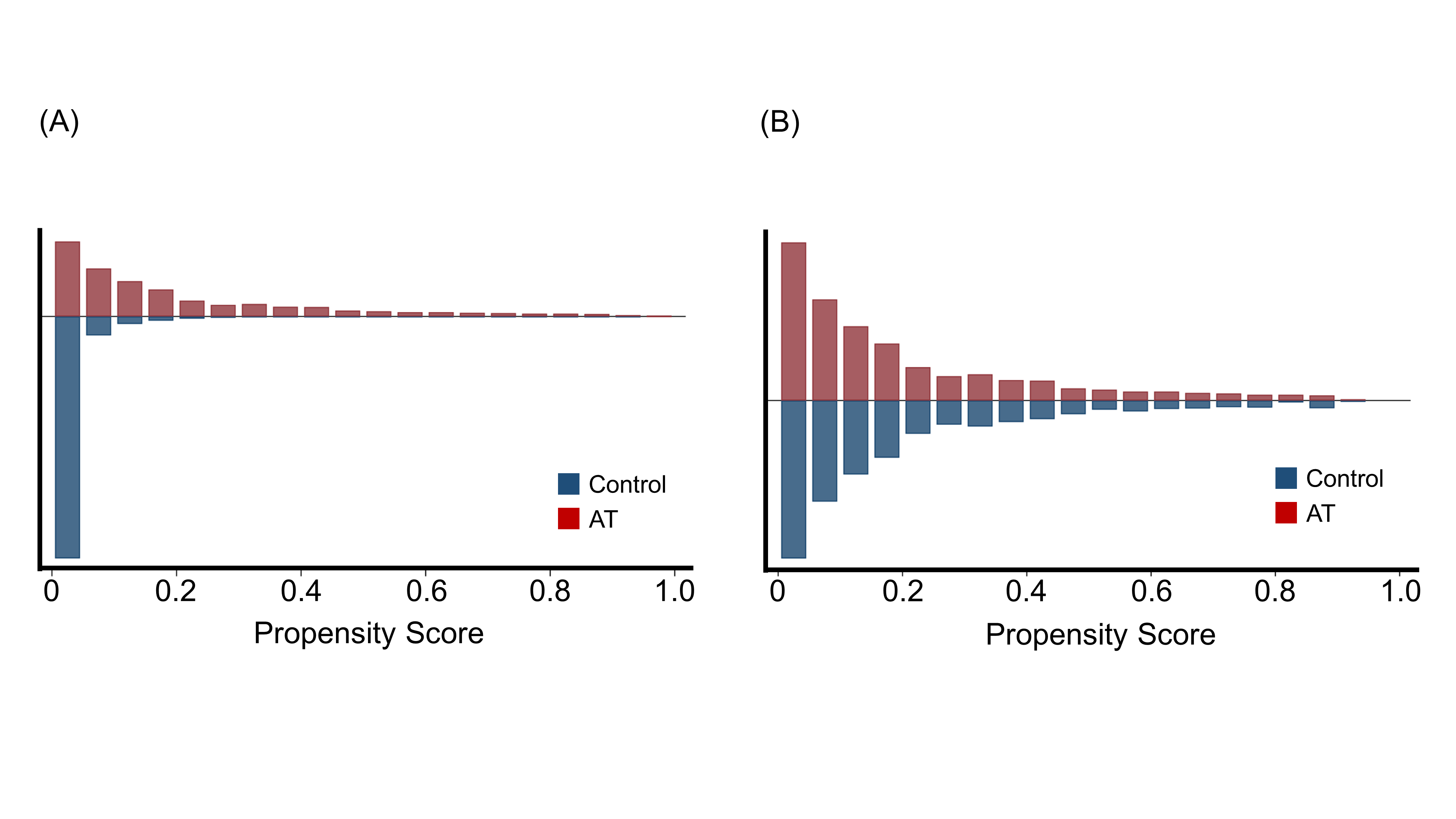

Supplement: Supplementary file 1 — Additional file 1: Figure S1. Distribution of propensity score (A) Before matching analysis (B) After matching analysis. AT, antithrombin. [file 12885_2020_7375_MOESM1_ESM.tif]
