# Supplementary material for: Antithrombin use and mortality in patients with stage IV solid tumor-associated disseminated intravascular coagulation: a nationwide observational study in Japan
Source: BMC Cancer. 2020 Sep 9;20:867. doi: 10.1186/s12885-020-07375-2 (PMC7488043; doi:10.1186/s12885-020-07375-2)
Supplement: Supplementary file 2 — Additional file 2: Table S1. ICD-10 codes and Japanese procedure codes for organ failure scores. [file 12885_2020_7375_MOESM2_ESM.docx]

**Table S1. ICD-10 and Japanese procedure codes to calculate organ failure scores**

| **Organ failure** | **ICD-10 codes** | **Japanese procedure codes or claims** |
| --- | --- | --- |
| **Cardiovascular** | I95 R57 | Vasopressor or inotrope (dopamine, dobutamine, epinephrine, norepinephrine, vasopressin) |
| **Respiratory** |  | Mechanical ventilation |
| **Neurologic** | F05 G93.4 |  |
| **Hematologic*** | D65 D69.5 D69.6 D69.8 D69.9 |  |
| **Hepatic** | K72.0 K76.3 |  |
| **Renal** | N17 | Renal replacement therapy |

ICD-10, International Classification of Diseases, tenth revision

*All patients diagnosed with disseminated intravascular coagulation (ICD-10 code: D65)
